# Implantation of INTERTAN™ nail in four patients with intertrochanteric fractures leading to single or comminute fractures: pitfalls and recommendations: a case series

**DOI:** 10.1186/1752-1947-8-383

**Published:** 2014-11-23

**Authors:** Yong Jiang, Jie Li, Hassan H Dib, Yuan-Cheng Li

**Affiliations:** 1 Department of Orthopaedics, First Affiliated Hospital of Dalian Medical University, 222 Zhongshan Road, Xigang District, Dalian 116011, Liaoning Province, China; 2 Health Services, Inuvik Regional Hospital, Beaufort-Delta Health & Social Services, Inuvik, North West Territory (NWT) X0E 0T0, Canada affiliated with University of British Colombia, Department of Rural Medicine, Vancouver, Canada

**Keywords:** Implantation difficulties, Intertrochanteric fracture, Intertain, China, Reaming, Obese, Older

## Abstract

**Introduction:**

Intraoperative technical complications are occasionally encountered while implanting INTERTAN™ nails for intertrochanteric fractures. Surgeons need to pay attention to the difficulties they may encounter during the implantation of an INTERTAN™ nail.

**Case presentation:**

We report four cases with intraoperative difficulties during the implantation of INTERTAN™ nails among Han Chinese patients from mainland China. In Case 1, during the operation on a 75-year-old woman, an anatomical specificity of excessive femoral shaft curvation at the coronal and sagittal planes was observed; a relatively smooth implantation was achieved by adjusting the entry point. In Case 2, due to fat obstruction, an INTERTAN™ nail was implanted at an oblique angle in 64-year-old obese woman, which resulted in an iatrogenic fracture of the proximal femur. In Case 3, an iatrogenic fracture of the distal femur developed in an 83-year-old woman because of violent hammering and underestimating of bone fragility. In Case 4, an iatrogenic fracture occurred in a 40-year-old woman around the distal locking slot during the drilling process.

**Conclusions:**

Preoperative evaluation should be considered as an important preparation for the implantation of an INTERTAN™ nail. Full-length anteroposterior and lateral radiographs of the injured femur are necessary to confirm the anatomical specificity. The vertical trajectory as well as sufficient reaming is important in reducing the possibility of iatrogenic fractures, particularly for obese patients. In older patients, violent hammering should be avoided and full reaming is recommended even if the canal seems to be wide enough. For cases where hard fracture reduction is predicted, the strategy of open reduction and fixation with a dynamic hip screw seems to be more rational and should be considered as an alternative method.

## Introduction

Operative treatment of intertrochanteric fractures were derived from the Gamma nail, passing through dynamic hip screw (DHS), proximal femural nail (PFN), proximal femural nail antirotation (PFNA), and progressed to the latest use of the TRIGEN INTERTAN nail [1-4]. With the unique integrated interlocking screw constructs, the TRIGEN INTERTAN nail provides all the benefits of a traditional antegrade intramedullary surgical nail approach as well as strengthening the stability and resistance to intraoperative and postoperative femoral head rotation. Furthermore, INTERTAN™ compression screw is always against the nail, which makes a medial migration impossible; thus, eliminating the Z-effect. In this report, we would like to illustrate the intraoperative pitfalls we have encountered while implanting INTERTAN™ nails in four patients who were admitted to our hospital.

## Case presentation

Our hospital department operated on 169 Han Chinese patients, with intratrochanteric fractures, from mainland China between Jan 2010-June 2013. The mean age of patients is 73 years old (data not shown). During the implantation of INTERTAN™ nail in patients with intratrochanteric fractures we encountered intraoperative difficulties. Some of these obstacles could be illustrated in the form of anatomical specificity of excessive femoral shaft curvation in the coronal and/or sagittal planes, fat obstruction, the use of oblique angle that resulted in iatrogenic fracture of the proximal femur; an iatrogenic fracture of the distal femur as a result of violent hammering; underestimating bone fragility; and iatrogenic fracture around the distal locking slot during the drilling process.

### Case 1

**Figure 1:**
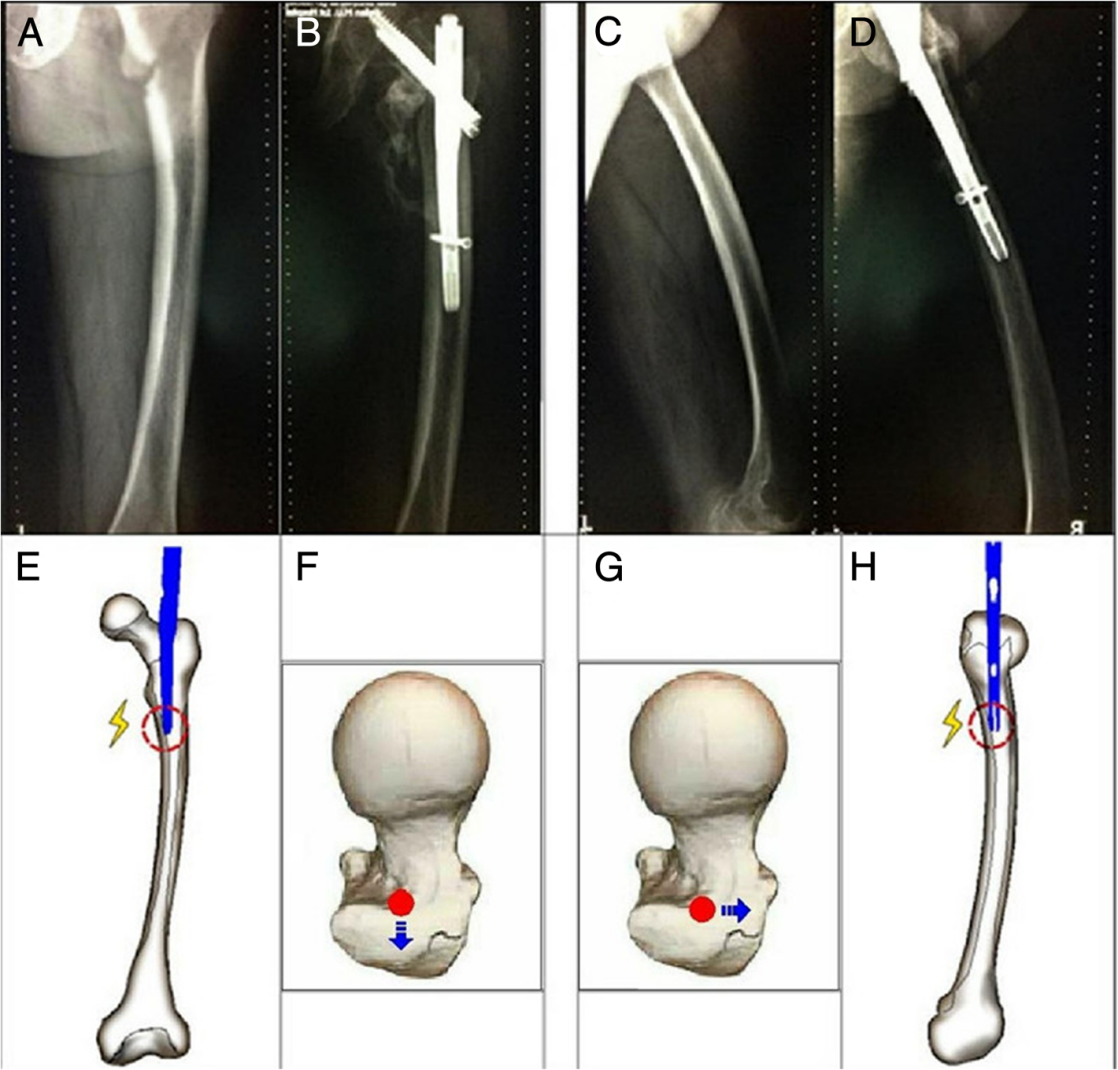
**Anterior, posterior, and lateral x-ray images of full length of the injured femur during & after two months surgery with femoral anatomical specificity of antecurvation. (A)** Full-length anteroposterior X-ray image of the injured femur during the operation. **(B)** Full-length anteroposterior X-ray image of the injured femur two months after surgery. **(C)** Full-length lateral X-ray image of the injured femur during the operation. **(D)** Full-length lateral X-ray image of the injured femur two months after surgery. **(E)** If the patient has the anatomical specificity of lateral curvation of the femoral shaft, resistance will be encountered while implanting the nail at the routine entry point. **(F)** Accordingly, the entry point should shift to the lateral. **(H)** If the patient has the anatomical specificity of antecurvation of the femoral shaft, resistance will be encountered while implanting the nail at the routine entry point. **(G)** Accordingly, the entry point should shift forward.

A 75-year-old Chinese Han woman, height: 160cm and weight: 55kg, presented with a simple pertrochanteric hip fracture below the lesser trochanter with a fracture classification AO: A1.3. A closed reduction with traction was achieved. While implanting an INTERTAN™ nail (INTERTAN™, 125°, 18.20-cm long, 10-mm diameter, Smith & Nephew, Andover, MA, USA) at the usual insertion point, we came across severe resistance. We tried to ream the medullary canal wide enough for insertion, but no relief was achieved. After careful analyses of the radiographs, during the intraoperative procedure, an excessive curvation of the femoral shaft was found in the coronal and sagittal planes. In response to those anatomical specificities, we adjusted the point of entry approach and reamed the medullar canal. With these improvements, a relatively smooth implantation was achieved (Figure 1).

### Case 2

**Figure 2:**
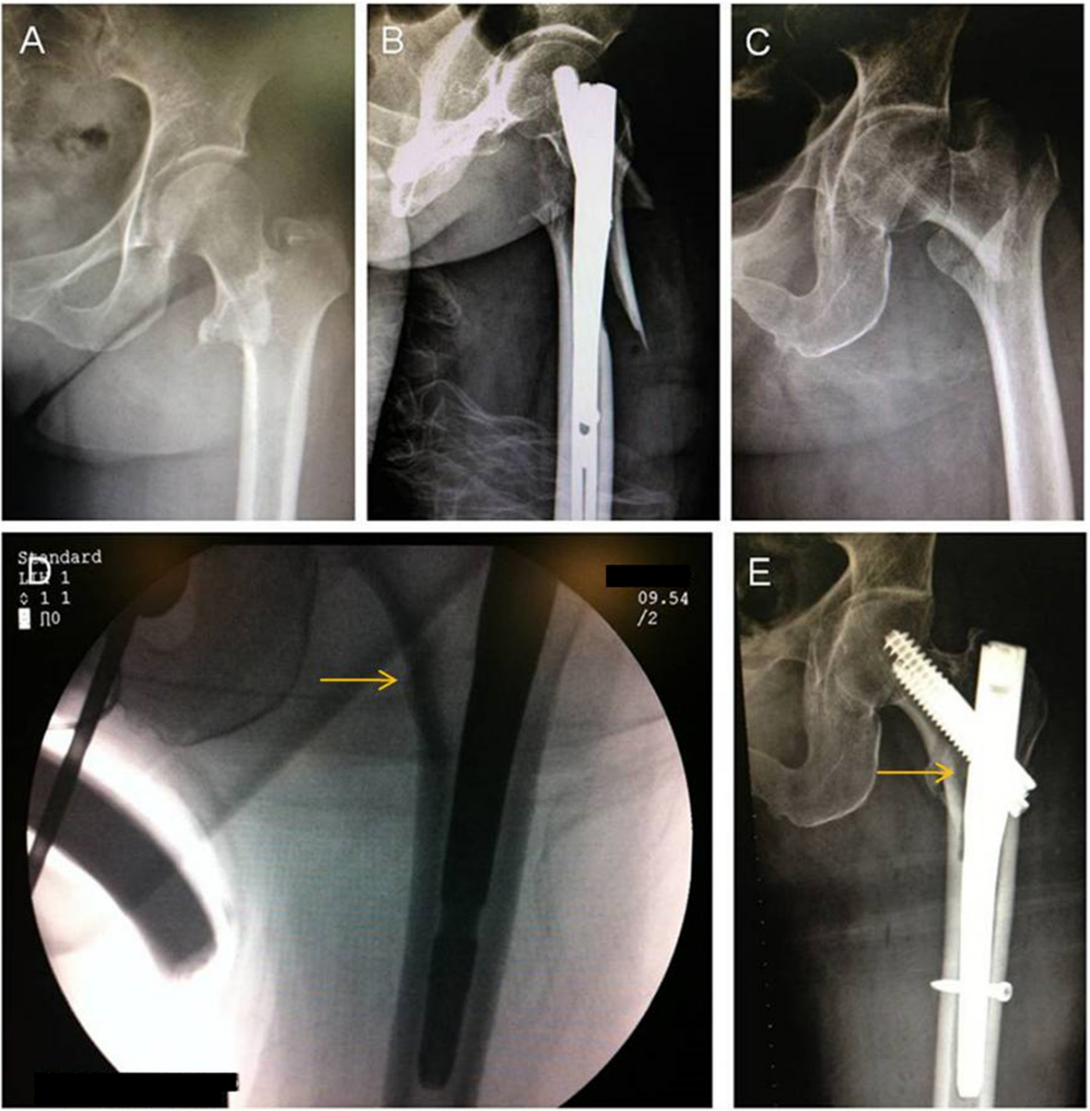
**Preoperative & postoperative anterior and posterior x-ray images of the injured hip with INTERTAN™ nails resulting in iatrogenic fractures in an obese patient then fixed with short and long INTERTAN™ nail. (A)** Preoperative anteroposterior X-ray image of the injured hip. **(B)** Postoperative anteroposterior X-ray image of the injured hip, showing the iatrogenic fractures of the proximal femur **(C)**. Preoperative anteroposterior X-ray image of the injured hip of another obese patient **(D)**. Based on our experience we kept the vertical trajectory with the nail insertion and fully reamed the medullar canal of the proximal femur, of the case in Figure 2A/B, with the anatomical specificity of an extremely narrow proximal canal (arrow). **(E)** With this improvement, a relatively smooth insertion was achieved (arrow).

A 64-year-old Chinese Han obese woman, height: 158cm and weight: 78kg, sustained a multifragmentary pertrochanteric hip fracture with two intermediate fragments (AO : A2.2). We opened the proximal femur and inserted a 12.5mm entry reamer to the greater trochanter. A less-than-ideal placement was achieved with the interlocking screw constructs. We tried to hammer the main pin deeper to get the perfect position for the interlocking screw constructs. During the procedure, an iatrogenic fracture of the proximal femur developed (Figure 2A, B). Based on our experience with obese patients we maintained a vertical trajectory approach during the nail insertion and fully reamed the medullar canal of the proximal femur, especially those with the anatomical specificity of an extremely narrow proximal canal (Figure 2C, D, E).

### Case 3

**Figure 3:**
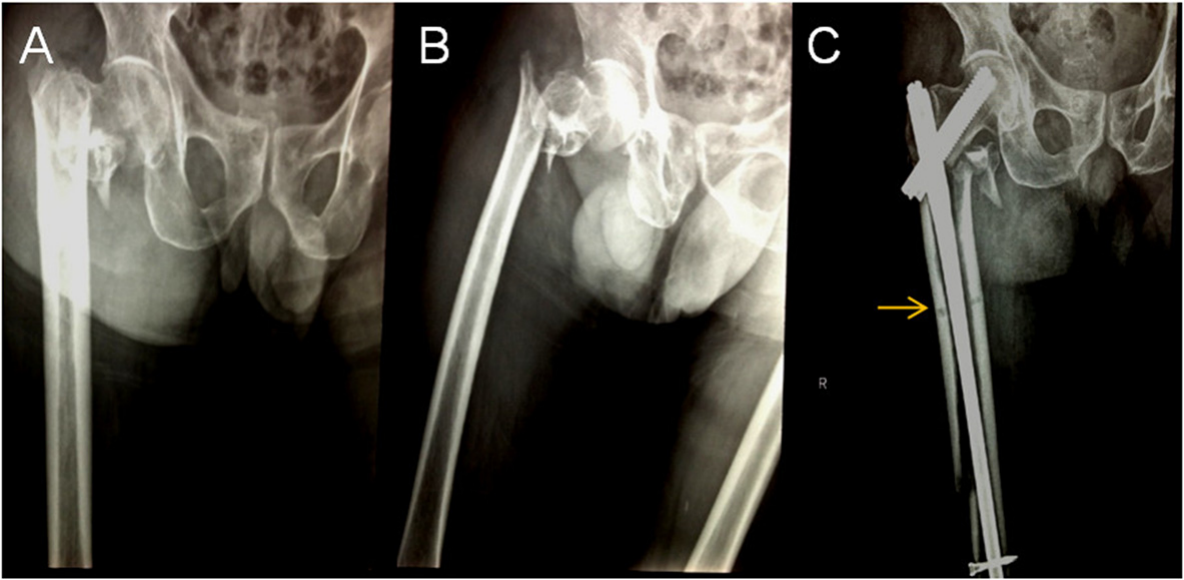
**Preoperative anterior, posterior, and lateral x-ray image of the injured hip fixed with INTERTAN™ nail resulted in an iatrogenic fracture then fixed with a long and short INTERTAN™ nail to fix the iatrogenic fracture. (A)** Preoperative anteroposterior X-ray image of the injured hip. **(B)** Preoperative lateral X-ray image of the injured hip. From the preoperative radiographs, the width of the proximal femoral canal was measured at around 11mm. **(C)** We did not expect there would be difficulties in the implantation of 10mm-diameter INTERTAN™ nail during the preoperative measurement of the femoral medullary cavity. After routine proximal canal reaming, we implanted the main pin manually and encountered resistance. We then hammered the main pin into the ideal position and performed the remaining steps. When the final X-ray was taken to confirm the position, an iatrogenic fracture of the distal femur was observed. The original short TRIGEN INTERTAN nail and the captured locking screw were removed (arrow). Subsequently, a longer INTERTAN™ nail was implanted to fix the iatrogenic fracture.

An 83-year-old Chinese Han woman, height: 177cm and weight: 70kg, sustained a multifragmentary pertrochanteric hip fracture with one intermediate fragment (lesser trochanter detachment) (AO of A2.1). After viewing the X-ray images during the preoperative measurement of the femoral medullary cavity, we did not expect there would be difficulties in implanting the INTERTAN™ nail (10mm in diameter) (Figure 3A, B). After a routine proximal canal reaming, we manually implanted the main pin and encountered resistance. We then hammered the main pin into the ideal position and performed the remaining steps. When the final X-rays were taken to confirm the positioning of the nail, an iatrogenic fracture of the distal femur was observed. The original short TRIGEN INTERTAN™ nail and the capturing locking screw were removed. Subsequently, a longer INTERTAN™ nail was implanted to fix the iatrogenic fracture (Figure 3C).

### Case 4

A 40-year-old Chinese Han woman, height: 175cm and weight: 72kg, sustained an intertrochanteric hip fracture (simple, oblique) (AO : A3.1). The reduction of the fracture was difficult due to comminuted fractures between the extended fragments. With the correction of multiple stresses by traction and manipulative reduction, as a result of the fractured fragments, the implantation of the main nail was inserted with difficulty. After the insertion of the integrated interlocking screw, we locked the distal slot. During the drilling, an iatrogenic fracture developed around the distal locking slot. An open reduction followed using a locking fixation plate to manage the femoral shaft fracture.

## Discussion

Proximal femoral fractures are common among older people requiring surgery for stable fixation and early ambulation. From the inception of DHS, such fixtures have become the gold standard for the treatment of intertrochanteric fractures [5-7]. In patients with stable fractures such devices produce excellent results. However, in patients with unstable fractures, the dynamic hip screw and plate are associated with an increased prevalence of complications such as cut-out, shaft medialization, shortening, and subsequent loss of reduction. For these reasons, there has been a sustained interest in the use of an intramedullary nail to treat proximal femoral fractures. However, the intramedullary method for trochanteric fractures requires extensive surgical experience. The incidence of complications, for example cut-out, femoral shaft fractures, and the steep learning curve has resulted, in the past, in the loss of popularity for these devices.

With the axial biomechanical advantages of the intramedullary nail, a series of intramedullary fixation implants evolved from the Gamma nail, PFN and PFNA to the latest TRIGEN INTERTAN nail. With the unique integrated, interlocking screw constructs, the TRIGEN INTERTAN nail provides all the benefits of a traditional surgical approach for the antegrade intramedullary nail, and also increases the stability and the resistance during the intraoperative and postoperative femoral head rotation. Furthermore, the INTERTAN™ compression screw is always against the nail so a medial migration is impossible; thus, eliminating the Z-effect. For the above reasons, the TRIGEN INTERTAN is technically an ambitious operative procedure for the treatment of intertrochanteric fractures. However, information about the intraoperative technical complications of implantation had hardly ever been reported.

When the first version of the Gamma nail was introduced to Asian patients, Leung *et al.*[8,9] did report problems about the geometric mismatch of the Gamma nail and the Chinese femur. Subsequently, a series of modifications in the design had improved it. Windolf *et al.*[10] also had reported that sufficiently deep insertion of the selected nail was impossible in three patients because of the anticurvation of the femoral shaft. In two of these cases, the entry point of the nail was placed too far dorsally. These problems were solved by changing to a thinner nail. As for Hwang *et al.*[11], they had reported the mismatch between the implant and the bowing of the femur in the coronal plane. During the insertion of the nail, an iatrogenic fracture developed along the medial cortex of the subtrochanteric area. As shown in Figure 1, our patient not only had the femoral anatomical specificity of anticurvation but also lateral curvation. After analyzing the radiographs during the operation, we changed the portal entry accordingly without changing to a smaller nail and then we placed the nail smoothly. As the person ages, the femoral diaphysis enlarges and the bowing of the femoral shafts increases [12]. The concern with using a straight intramedullary nail in a bowed osteopenic femur can result in the impinging of the main nail, and in some cases even perforating the distal femoral metaphyseal cortex. Additionally, when the nail engulfs the femoral cortex, any locking screws placed in the distal part of the femur may cause a stress riser in this area, which might lead to an iatrogenic fracture or pain in the postoperative rehabilitation period [13]. It is difficult to perceive the bowing of the femoral shaft with AP and lateral views of the injured hip. Based on our experience, full-length AP and lateral radiographs of the injured femur are necessary for the confirmation of anatomical specificity. With this evaluation, potential intraoperative complications during implantation should come to the surgeon’s attention as well as the correspondent shift of portal entry (Figure 1). If excessive curvation of the femoral shaft in the coronal and sagittal planes is observed before the operation, then the strategy of open reduction and fixation with a DHS seems to be more rational and should be considered as an alternative method.Based on our experience with one of the cases, that is our obese patient (Figure 2A, B), we believe that it is important to achieve a vertical trajectory approach with the nail insertion; this can be difficult in obese patients. If the intramedullary nail is inserted at an oblique angle due to fat obstruction, the nail itself can impact the relatively soft bone of the lateral aspect of the greater trochanter, which could result in an iatrogenic fracture of the proximal femur. It is better to perform a vertical trajectory approach with the nail insertion; thus, a jackknife position is the preferred one in our hospital. For the case in Figure 2C, D, E when stiff resistance was encountered, we had to adjust the reamer assembly to the desired trajectory. Moreover, by utilizing the intraoperative radiographs, we had observed the anatomical specificity of the narrow proximal femoral canal that enabled us to thoroughly ream the medial cortex. With this improvement, a relatively smooth insertion was acquired. As for our patient in Figure 3A, B, the width of the proximal femoral canal was measured at around 11mm from the preoperative radiographs. We underestimated the difficulties of implants due to the measured width and the predicted osteoporosis in our 83-year-old patient. In this case, the resistance had developed for several reasons, most likely: (1) there was a less-than-ideal portal entry and (2) the trajectory resulted in the collision between the tip of the nail and the cortex in the process of moving forward. After that, iatrogenic fractures of the distal femur developed because of the violent hammering and underestimating of the bone fragility.

As described in Case 4, our patient had suffered comminute intertrochanteric fractures where there was significant shift in the fragments. To stretch the muscle that was attached to the bone, it was extremely hard to reduce the fragments and insert the nail simultaneously into the distal femoral canal - open reduction and implantation took place. After the insertion of the integrated interlocking screw, we proceeded in locking the distal slot. During the drilling, an iatrogenic fracture around the distal locking slot developed. We analyzed that the huge stress from the separated fragments on the nail resulted in the deviation of the nail and serious tension on the cortex was produced due to the impact of the distal nail. When there is serious tension, especially when the drilling point is so close to the edge, the distal cortex broke while drilling it. If serious tension had impacted on the cortex - developed due to deviation of the INTERTAN™ tip - more attention should have been paid to avoid iatrogenic fractures. Reverse rotation and gentle manipulation during drilling are recommended. Furthermore, we believe that if hard reduction is predicted from the preoperative evaluation, the strategy of open reduction and fixation with a DHS seemed to be more rational and/or should be prepared as an alternative method. In spite of the biomechanical disadvantages, a DHS has a series of advantages such as low blood loss, a shorter operation time and more satisfactory reduction in these cases [14].

## Conclusions

The following recommendations should be considered in the nailing of the pertrochanteric fractures. Preoperative evaluation should be considered as an important preparation for INTERTAN™ implantation. Full-length AP and lateral radiographs of the injured femur are necessary for the confirmation of anatomical specificity; vertical trajectory as well as sufficient reaming of the medullar canal of the proximal femur is important to reduce the possibility of iatrogenic fractures to ensure smooth insertion of the main pin, particularly in obese patients. Violent hammering should be avoided and full reaming is recommended in older patients even if the canal seems to be wide enough. For cases where hard fracture reduction is predicted in the preoperative evaluation, the strategy of open reduction and fixation with a DHS seems to be more rational and should be prepared as an alternative method.

## Consent

Written informed consent was obtained from the patients for publication of this case report and any accompanying images. A copy of the written consent is available for review by the Editor-in-Chief of this journal.

## Abbreviations

AO: classification of fractures; A1: simple (two fragment) pertrochanteric factures; A1.3: hip fracture: A1.3 fractures, where the fractures are below the lesser trochanter; A2: multifragmentary pertrochanteric; A2.1: with one intermediate fragment (lesser trochanter detachment); A2.2: with two intermediate fragments; A3: intertrochanteric fractures; A3.1: intertrochanteric fracture-simple, oblique; AP: anteroposterior; DHS: dynamic hip screw; PFN: proximal femoral nail; PFNA: proximal femur nail antirotation.

## Competing interests

The authors declare that they have no competing interests.

## Authors’ contributions

JY wrote the initial draft, performed field work, collected the data and participated in the statistical analysis. LJ performed field work, collected the data and participated in the statistical analysis. LYC designed the study and supervised the study. HHD analyzed the data and guided and participated in writing and finalizing the manuscript. All authors read and approved the final manuscript.
